# Modeling Human Brain Tumors and the Microenvironment Using Induced Pluripotent Stem Cells

**DOI:** 10.3390/cancers15041253

**Published:** 2023-02-16

**Authors:** Zahraa I. Khamis, Drishty B. Sarker, Yu Xue, Nancy Al-Akkary, Viviana D. James, Changchun Zeng, Yan Li, Qing-Xiang Amy Sang

**Affiliations:** 1 Department of Chemistry and Biochemistry, Florida State University, Tallahassee, FL 32306, USA; 2 Department of Industrial and Manufacturing Engineering, FAMU-FSU College of Engineering, Florida State University, Tallahassee, FL 32310, USA; 3 High-Performance Materials Institute, Florida State University, Tallahassee, FL 32310, USA; 4 Laboratory of Cancer Biology and Molecular Immunology, Department of Biochemistry, Faculty of Sciences-I, Lebanese University, Beirut, Lebanon; 5 Department of Chemical and Biomedical Engineering, FAMU-FSU College of Engineering, Florida State University, Tallahassee, FL 32306, USA; 6 Institute of Molecular Biophysics, Florida State University, Tallahassee, FL 32306, USA

**Keywords:** human brain cancer, induced pluripotent stem cell technology, tumor microenvironment, isogenic cells, three-dimensional (3D) cell culture models, brain organoids, disease modeling, drug screening and development

## Abstract

**Simple Summary:**

Induced pluripotent stem cells (iPSCs) are crucial for disease modeling and cell-based therapy because they serve as an infinite source of specific human cell types. The use of iPSCs in cancer immunotherapy and cell transplantation therapy has garnered much attention in personalized medicine. To improve the efficacy and specificity of brain cancer treatment, iPSCs can be used to derive human brain tumor models for therapeutic evaluation. This review summarizes the utilization of human iPSCs to generate brain-specific cells, organoids, and tumor models for brain cancer modeling and drug testing. In addition, current challenges, limitations, and future prospects to find a more efficacious approach for treating human brain cancers are discussed.

**Abstract:**

Brain cancer is a group of diverse and rapidly growing malignancies that originate in the central nervous system (CNS) and have a poor prognosis. The complexity of brain structure and function makes brain cancer modeling extremely difficult, limiting pathological studies and therapeutic developments. Advancements in human pluripotent stem cell technology have opened a window of opportunity for brain cancer modeling, providing a wealth of customizable methods to simulate the disease in vitro. This is achieved with the advent of genome editing and genetic engineering technologies that can simulate germline and somatic mutations found in human brain tumors. This review investigates induced pluripotent stem cell (iPSC)-based approaches to model human brain cancer. The applications of iPSCs as renewable sources of individual brain cell types, brain organoids, blood–brain barrier (BBB), and brain tumor models are discussed. The brain tumor models reviewed are glioblastoma and medulloblastoma. The iPSC-derived isogenic cells and three-dimensional (3D) brain cancer organoids combined with patient-derived xenografts will enhance future compound screening and drug development for these deadly human brain cancers.

## 1. Introduction

Early embryonic development marks the division and transformation of a totipotent zygote into pluripotent cells that can give rise to all cell types of the body [1]. The induction of pluripotency in somatic cells is of enormous scientific interest and is a powerful tool for medicine and basic biological research [2]. Before the advent of culture-induced reprogramming, the production of pluripotent cells was mostly achieved using exposure-based approaches such as somatic cell nuclear transfer (SCNT), cell fusion, or reprogramming by cell extracts [3]. Induced pluripotent stem cell (iPSC) technology later emerged as the most viable option for pluripotent stem cell production due to its simplicity and reproducibility [4]. The pioneering work of Takahashi and Yamanaka in 2006, which reprogrammed mouse somatic cells into pluripotent stem cells by introducing a specified set of transcription factors, was a breakthrough in iPSC derivation [5]. This discovery subsequently fueled other studies to generate iPSCs from different human somatic cells using defined factors [6,7,8]. Human iPSC production from skin or blood cells represents an abundant source of human cell types needed for therapeutic purposes. Hence, iPSC reprogramming is now one of the most prominent trends in basic research fields and in medicine. It is applied in disease modeling, drug discovery [9,10,11], and cell therapy for the treatment of different kinds of diseases, such as retinal diseases [12,13], neurodegenerative diseases and injuries [14,15,16], thrombocytopenia [17], and cancer [18,19].

Models using iPSCs are compelling alternatives to animal models for studying neural disorders and brain tumors because iPSC-derived neurons possess similar genetic information, cell structure, and neuronal connections as those found in the human brain. Pathological models are now available for major neurological disorders that have been used to elucidate pathogenic mechanisms and to evaluate drugs preclinically [10,20,21]. However, brain tumor modeling using iPSCs is still a challenging and underexplored area of research.

Despite appreciable improvement in cancer survivorship, brain cancers continue to show poor prognosis, largely attributable to the absence of suitable brain tumor models that limit the study of complex brain tumor biology and drug development. Glioma and medulloblastoma are two primary brain tumors with the highest incidence in adults and children, respectively [22]. The most aggressive glioma variant, glioblastoma, frequently develops therapeutic resistance to hand patients a scanty median survival of 15 months [10,22,23,24]. Medulloblastoma, which accounts for 20% of all childhood brain malignancies [25,26], shows a better response to radiation and chemotherapy. However, the search for medulloblastoma treatment modalities without long-term adverse effects is ongoing [22]. The current scenario implies that obtaining reliable glioma and medulloblastoma models would be the key to understanding tumor biology and finding effective treatment strategies to enhance brain cancer survival.

The past decade has seen a gradual emergence of iPSC-derived human brain tumor platforms [10,27,28,29] resulting from the recent development of methods for capturing tumorigenic mutations and organoid formation. The advent of customizable and highly efficient genome editing technologies like CRISPR has made brain tumor modeling plausible, since engineered systems offer targeted study of patient-specific diseases with germline or somatic mutations [30]. Apart from tumor cells, modeling blood vessels and other supporting cells of the brain tumor microenvironment is also of paramount interest as they affect how tumor cells respond to stress and develop therapeutic resistance [24]. Therefore, creating a faithful brain tumor avatar with all its essential components is crucial for predicting drug response and improving treatment outcomes for dreadful brain malignancies [23].

In this review, we discuss strategies for obtaining specific brain cells and brain organoids from iPSCs with a special focus on brain tumor models—their construction and usefulness in shaping brain cancer research.

## 2. In Vitro Modeling of the Human Brain with Induced-Pluripotent Stem Cells

Since their discovery, iPSCs have enabled unprecedented advancements in studying human brain physiology and pathobiology. Researchers have successfully used iPSCs to generate various types of brain cells in 2D cultures to model specific brain regions in 3D organoid systems and to restructure the blood–brain barrier (BBB) in vitro. Collectively, these models contributed to the generation of a brain-like organ that may recapitulate the architecture of a developing human brain.

### 2.1. Deriving Brain Microenvironment Cells Using iPSCs

**Figure 1:**
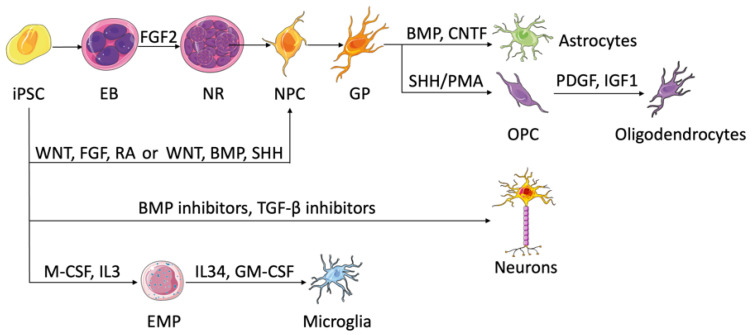
Brain cells and their differentiation from iPSCs. NPCs exist in neural rosettes and develop glial phenotypes spontaneously, which can differentiate into astrocytes by the addition of STAT pathway activators, such as BMP and CNTF. Following SHH or PMA addition, NPCs can turn into oligodendrocytes. PDGF and IGF1 are used in the process of maturation. NPCs can also come from iPSCs directly. Wnt, FGF, and RA establish anterior–posterior identities of NPCs, while Wnt, BMP, and SHH form dorsal–ventral identities. Neuron formation from iPSCs requires dual SMAD signaling inhibition using BMP and TGF-β inhibitors. Myelopoiesis takes place upon the addition of M-CSF and IL3. EMPs generate microglia once treated with IL34 and GM-CSF. EB, embryonic body; NR, neural rosette; NPC, neural progenitor cell; GP, glial phenotype; OPC, oligodendrocyte progenitor cell; EMP, erythromyeloid progenitor.

The human brain consists of two major cell types—the neurons and the glial cells—that work in concert to ensure the proper execution of its functions. While the neurons are the primary cells responsible for signal transmission and processing, the glial cells, composed of astrocytes, oligodendrocytes, and microglia, support various neuronal functions and play active roles in synaptogenesis and brain development [31]. These brain-specific cells can be differentiated in vitro from iPSCs in 2D monolayer cultures (Figure 1). Despite their inherent advantages, the 2D culture models fail to properly mimic the complex structure of the human brain, ranging from cell–cell and cell–matrix interactions to cell polarity and migration [32].

#### 2.1.1. Neural Progenitor Cells and Neurons

Neural progenitor cells (NPCs) serve as precursor cells that can differentiate into neurons, astrocytes, and oligodendrocytes, so the generation of NPCs is considered substantial for the study of neurodevelopment and neurological diseases. With the advent of iPSCs, various protocols have been developed to differentiate iPSCs into NPCs [33]. Historically, this differentiation involves the spontaneous formation of small spheres called embryoid bodies (EB) if culturing iPSCs in suspension, which after the addition of specific growth factors such as fibroblast growth factor-2 (FGF-2) would generate neural tube-like structures called neural rosettes [34]. The cells of the neural rosettes have characteristics similar to embryonic neuroepithelia and mainly contain NPCs [33]. However, the variety in iPSC lines and experimental setups created variations in differentiated NPCs and urged the search for an alternative [35]. In 2009, Chambers et al. established a protocol for neural induction that involves dual inhibition of SMAD signaling using bone morphogenetic protein (BMP) pathway inhibitors and transforming growth factor beta (TGF-β) pathway inhibitors [36]. This dual-SMAD inhibition protocol circumvented the EB formation and its associated variability in monolayer cultures, as well as induced efficient differentiation in a short period of time. Nowadays, various protocols for neural differentiation of iPSCs are available with slight variations from the aforementioned methods.

It is important to note that NPCs can be generated from iPSCs as region-specific subtypes based on the available morphogens. While wingless/integrated (Wnt), FGF, and retinoic acid (RA) morphogens generate progenitors along the anterior–posterior axis, exposure to Wnt, BMP, and sonic hedgehog (SHH) results in cells with dorsal–ventral identities (reviewed by Tao et al.) [37]. In addition to regional patterning, one can enrich certain neural progenitors by exploiting the intrinsic time course needed for the production of different progenitor subtypes. Mitogens and γ-secretase inhibitors are combined to promote the rise of target progenitors and keep them in the cell cycle, which enriches the population of target progenitor cells by preventing them from differentiating to the next stage. Cultured in a neurotrophic factor-rich medium, regionally patterned neural progenitors can give rise to various neuronal subtypes such as dopaminergic, glutamatergic, GABAergic, and cholinergic. When engrafted into the mouse brain, neuron subtypes appear to localize in different areas, following functional maturation dictated by their locally intrinsic programs that have not been fully understood but take a long time, more than 6 months in certain cases [33,37]. The availability of in vitro human iPSC-derived neurons enables in-depth studies of neurological diseases that are caused by damage in specific neural subtypes.

#### 2.1.2. Astrocytes

Differentiation of iPSCs into astrocytes takes a prolonged time and occurs in three steps: (1) neural induction of iPSCs into NPCs through EB formation, (2) expansion of NPCs until they acquire a glial phenotype, and (3) differentiation of glial progenitors into astrocytes driven by several factors, such as BMP and ciliary neurotrophic factor (CNTF), which activate the signal transducer and activator of transcription (STAT) pathway [37,38]. Neural progenitors patterned to different areas of the brain are found to switch to glial cells and then to astrocytes while retaining their regional identities [39]. While modeling astrocytes, the iPSC–astrocytic derivatives should recapitulate not only the structural but also the physiological features of genuine astrocytes [40].

#### 2.1.3. Oligodendrocytes

Involved in axonal myelination, oligodendrocytes emerge late during normal development and follow an intricate spatiotemporal specification. Similar to astrocytes, oligodendrocytic differentiation from iPSCs begins with NPC generation followed by gliogenesis [41,42]. The transition of the gliogenic precursors into oligodendrocyte progenitor cells coexpressing oligodendrocyte transcription factor 2 (Olig2) and NK2 homeobox 2 (NKX2-2) is maintained by the addition of SHH or its agonist purmorphamine (PMA) [43]. The mature oligodendrocytes are finally established with the help of growth factors such as platelet-derived growth factor (PDGF) or insulin-like growth factor 1 (IGF1) [37,44]. Since the differentiation time of the previous protocols is too long, efforts are focused on accelerating the process. Studies show that overexpression of oligodendrocyte-specific transcription factors, such as SPY-box transcription factor 10 (SOX10), can induce fast and efficient differentiation of iPSCs into oligodendrocytes capable of myelinating axons [44,45]. Recently, Shaker et al. developed a protocol that generated myelinating human oligodendrocytes in only 42 days, using an iPSC line that overexpresses SOX10 [46].

#### 2.1.4. Microglia

Microglia are the resident macrophages of the central nervous system (CNS) and the primary gatekeepers against infections. Aside from their role in innate immunity, microglia are critical players in neurological diseases, highlighting the need for the development of in vitro human-like microglia models that can recapitulate their in vivo counterparts. Recent protocols for iPSC-derived microglia rely on the latest developmental studies that trace microglia lineage to erythromyeloid progenitors (EMPs) in the yolk sac [47]. In most studies, myelopoiesis of iPSCs is induced by the addition of macrophage colony stimulation factor (M-CSF) and interleukin 3 (IL3) in the culture medium, where pure microglia precursors occur in the supernatant persistently. For the maturation of microglia, IL34 and granulocyte–macrophage colony-stimulating factor (GM-CSF) are needed. Additional factors, such as stem cell factor (SCF), vascular endothelial growth factor (VEGF), and BMP4, are optional and can be added for enhancing differentiation efficiency. While the differentiated cells exhibited a plethora of microglia structural and functional features compared to primary microglia culture, additional studies are required to validate the results with in vivo microglia [48].

### 2.2. Derivation of Brain Organoids from iPSC

**Figure 2:**
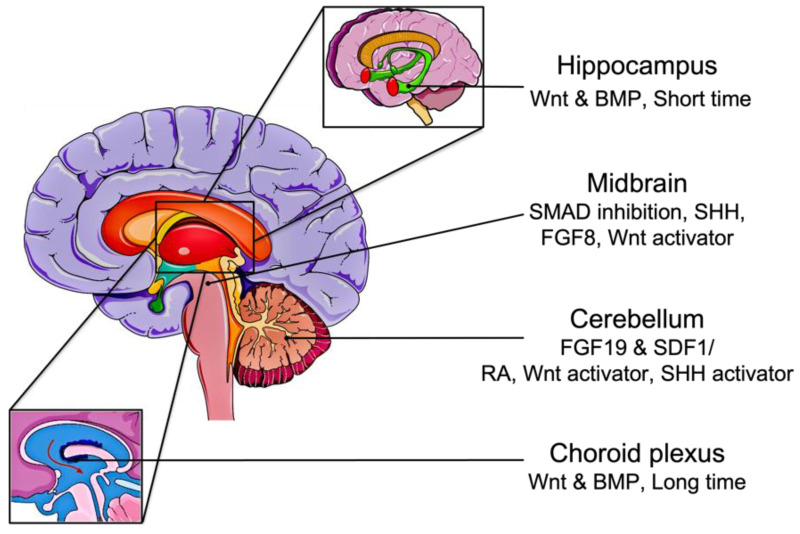
Brain organoid differentiation from iPSCs. The addition of RA, Wnt, and SHH activators generates cerebellar organoids directly from iPSCs. Pluripotent stem cells can be differentiated into cerebellar tissue through FGF19 and SDF1 treatment. Induced PSCs aggregate into embryoid bodies in serum-free suspension. Short-term treatment of Wnt and BMP to EBs develops hippocampus organoids, while long-term treatment generates choroid plexus organoids. If treated with FGF19 and SDF1, EBs can form cerebellum organoids.

To more accurately model the human brain, a 3D culture system is required. These 3D structures, also termed organoids, consist of organ-specific progenitor cells capable of spontaneous self-organization and differentiation into various tissue-specific cells that can mimic some of the organ’s structural and functional features [49]. We reviewed using specific factors to achieve corresponding brain organoids from iPSCs or iPSC-derived EBs (Figure 2). In 2008, iPSCs were shown to self-aggregate into EBs when grown in serum-free floating culture, generating neural tissue with apicobasal polarization and cortical fate [50,51]. Another revolution in brain organoids that directed the differentiation of EBs into various brain regions was the introduction of *Matrigel*, an extracellular matrix derived from a mouse sarcoma cell line, into the culture [52]. When stabilized in *Matrigel*, EBs spontaneously developed into optical cup characteristics of retinal tissue [53]. Later in 2013, Lancaster et al. produced cerebral organoids by embedding EB-derived neuroectodermal tissue in *Matrigel* and then culturing the complex in differentiation media with RA supplement [54]. This protocol generated multiple brain regions and heterogeneous cytoarchitecture. However, the cellular diversity of iPSCs, the inherent variability among different batches, and the lack of reproducibility raised concerns about the relevance of this organoid model for applications in disease modeling and drug testing.

Alternatively, other methods have been described to restrict the differentiation of the organoids into specific brain regions. By adding defined signaling molecules, organoids can be patterned into forebrain, midbrain, or hindbrain. For example, the addition of SMAD inhibitors can guide the organoids into dorsal forebrain fate, and if followed by SHH agonists, can promote ventral forebrain identities [55]. Furthermore, treatment of EBs with Wnt and BMP for a short period of time develops hippocampal organoids, while prolonged exposure generates choroid plexus organoids [56]. Midbrain regions are established by the addition of SHH, FGF8, and a Wnt activator to the culture medium after SMAD inhibition [57]. The most elaborate hindbrain structure, the cerebellum, was also formed in vitro using FGF19 and stromal cell-derived factor 1 (SDF1) [58]. Hua et al. demonstrated the formation of cerebellar organoids from iPSCs using the combination of RA, Wnt activator, and SHH activator [59,60].

Even though the addition of external factors has patterned the brain organoids into specific regional identities, these regions lack proper morphogen spatiotemporal gradients, functional connectivity among diverse brain regions, and non-ectodermal cell lineages, such as microglia and endothelial cells (ECs). This can substantially affect brain organoid development, resulting in an unfaithful replica of the human brain [50].

A multitude of studies has emerged to address the aforementioned shortcomings of the patterning protocols. Cederquist et al. engineered a pseudo-signaling center by embedding inducible SHH-expressing iPSCs at one pole of a developing organoid generating forebrain topography reminiscent of in vivo neurodevelopment [61]. Additionally, Rifes et al. implemented a microfluidic approach to create a Wnt-activating gradient that can induce neural tube regionalization [62]. To optimize neural connectivity, assembled organoids are formed by developing individual brain regions first and subsequently fusing them in a coculture. Several studies have reported the fusion of ventral and dorsal forebrain organoids, which resulted in dorsal migration of the interneurons and their integration into cortical microcircuits characteristics of the in vivo process [63,64,65]. More recently, Xiang et al. fused cortical and thalamic organoids to model the reciprocal thalamocortical projections that occur in the human brain [66]. Additionally, the incorporation of microglia into brain organoids that lack an innate source is essential due to their vital roles in neuronal development and immune response regulation. However, Ormel et al. showed that brain organoids developed without dual-SMAD inhibition could spontaneously give rise to mesodermal progenitors and later microglia-like cells. These microglia-like cells exhibited some phenotypes and functions typical of adult microglia [67]. The derived isogenic microglia-like cells were also shown to express phenotypic markers, phagocytose particles, and secrete inflammatory cytokines, as well as the ability to integrate with the dorsal and ventral forebrain organoids [68]. As the organoid grows, vascularization becomes essential to ensure proper delivery of nutrients and oxygen to the core. Recently, Ham et al. reported the generation of vessel-like structures with mature BBB features upon the treatment of cerebral organoids with VEGF [69]. Alternative methods employed to prevent necrosis in the organoid core include transplantation of the organoid in the mouse cortex [70] or growing the organoid as slice cultures [71].

Collectively, despite various pitfalls, these breakthroughs in brain organoid development from human iPSCs have provided unprecedented opportunities to understand and manage brain development and neurological disorders.

### 2.3. Induced PSC-Derived Blood–Brain Barrier Models

The blood–brain barrier (BBB) is an exceptionally selective barrier with rich composition of unique brain microvascular endothelial cells (BMECs) and other supporting cells (neurons, astrocytes, and pericytes) of the neurovascular unit (NVU). Through its dynamic cellular complexes and tight junctions, the BBB is involved in the maintenance of brain homeostasis and the regulation of any interchange between the bloodstream and the CNS [72]. BBB components express specific molecular transporters controlling the entry of molecules and nutrients into the brain [73]. Through this, the BBB is at the forefront of ensuring optimal CNS function by moderating the influence of systemic fluctuations and harmful substances (e.g., pathogens and toxins) on the brain [74].

Specialized endothelial cells known as BMECs compose the innermost layer of the BBB’s physiological barrier. Alteration of these cells on a structural and functional level is linked to neurological disorders such as traumatic brain injury, Alzheimer’s disease, and Parkinson’s disease [75,76]. Pericytes are multifunctional cells found inside capillaries that also constitute an important part of the NVU. With their stem cell-like properties, they are involved in several physiological processes, including regulation of the BBB, neuroinflammation, vasculogenesis, and angiogenesis. Disruption of pericyte function is also associated with brain pathologies, particularly vascular diseases [77].

In a pioneering approach to generate BMECs from iPSCs, Lippmann et al. employed endothelial and neural codifferentiation of iPSCs in an unconditioned medium followed by expansion of the cells in an endothelial cell medium and selective matrix-aided cell purification. By coculturing these iPSC-derived BMECs (iBMECs) with astrocytes, the group was able to induce the expression of endothelial transporters and receptors, in addition to the recreation of BBB attributes such as well-organized tight junctions and polarized efflux transporter activity [78]. The key steps of endothelial induction and BMEC specification and purification were adopted later in the iBMEC protocols reviewed by Workman and Svendsen [79]. The successful protocols resulted in optimized iPSC seeding density [80], faster differentiation [81], accurate developmental course [82,83], simplified defined media [84], increased iBMEC purity [85], and capacity for cryopreservation [86,87]. However, limitations of the iBMEC protocols still exist and mainly include the generation of a homogeneous epithelial cell population. It is critical for disease pathology and drug screening research to generate endothelial cells conforming as much as possible to their in vivo counterparts at both the transcriptional and the functional levels. To that end, reprogramming the epithelial lineage of BMECs into vascular ECs can be achieved by overexpressing endothelial transcription factors ETV2, ERG, and FL1 [88]. Regarding the acquisition of canonical BBB characteristics (i.e., expression of GLUT1, increased claudin-5, decreased PLVAP, and decreased permeability) by iBMECs, Gastfriend et al. showed in a recent study that activation of Wnt signaling is essential to express the endothelial phenotypes [89].

Pericytes have two distinct origins and can be generated from iPSCs through either mesoderm or neural crest induction [90]. Mesoderm induction can be achieved in several ways, one of which involves the addition of BMP4, activin A, and VEGF [91,92]. Alternatively, multipotent vascular progenitor (MesoT) cells can be generated from iPSCs and differentiated into pericytes by mesoderm induction [93]. Neural crest induction of iPSCs into pericytes relies on canonical Wnt signaling activation in a protocol similar to endothelial cell differentiation [94]. Wnt activation and simultaneous activin/nodal/BMP/TGF-β1 inhibition improve the faithfulness of in vitro BBB models [95]. It is thus evident that neurovascular functions greatly depend on key signaling pathways and interactions between brain-specific pericytes and surrounding cells, such as endothelial cells, astrocytes, and neurons. These pathways include PDGF-BB, TGF-β, and Notch signaling. Specifically, pericyte survival is promoted by PDGF-BB-PDGFRb signaling, while pericyte attachment to endothelial cells is mediated by TGF-β signaling [96]. Notch signaling plays a crucial role in vascular development and arteriovenous specifications [96].

Coculture of iBMECs with pericytes, astrocytes, and neurons is an attractive approach to enhance BBB properties in models, including transendothelial electrical resistance (TEER) and low passive permeability [97,98,99]. This has led to the generation of iPSC-derived NVU supporting cells and combining them with iBMECs for an isogenic model of human BBB [95,100,101,102]. Such models should allow a deeper understanding of physiological processes in both healthy and diseased states. Recently, Marzano et al. showed that brain pericytes and endothelial cell coculture derived from iPSCs showed appropriate inflammatory responses to amyloid-β42 oligomers [103]. Similar observations were made for iPSC-derived astroglial cells [104]. These studies warrant effective functionalization of cells toward reliable BBB modeling.

In vitro models derived from human iPSCs proved to be important tools for research considering interspecies variations in BBB receptor expression and the difficulty of using fresh tissue from human biopsies [105,106]. While 2D models were initially promising, the incorporation of other relevant factors, such as cell–cell interactions and fluid shear stress remained necessary to ensure EC and pericyte function [95,107]. This limitation was addressed by incorporating microfluidic channels synthesized from biomaterials [108] and including several iPSC-derived cell types in the BBB chips [109]. Through the generation of a 3D environment, such as BBB chips and iPSC-derived 3D brain organoids, observation of multilineage differentiation, self-organization into heterogeneous cell populations, and neurodevelopmental/neurodegenerative gene regulation patterns becomes possible [110]. Flow dynamics and BBB–brain tissue interactions can therefore be readily explored, yielding a better understanding of disease pathology and providing a physiologically relevant platform for disease modeling and drug screening.

## 3. Induced Pluripotent Stem Cell-Derived Brain Tumor Models

Powered by their proclivity to self-renew and differentiate into specific cell lineages, iPSCs offer a valuable and easily manipulable model system that can be used to understand brain cancer progression, screen for anticancer drugs, and develop personalized therapeutic approaches. Primary brain tumors arising within the brain mainly consist of gliomas and medulloblastomas, which occur in adults and children, respectively. Diffuse gliomas, the most common primary tumors of the brain, include low-grade gliomas, astrocytomas and oligodendrogliomas, and high-grade glioblastomas that are distinguished from one another by the mutational status of isocitrate dehydrogenase (IDH) genes. IDH mutations are enriched in low-grade gliomas compared to glioblastomas, and thus infer a strong positive prognostic marker [111]. While the generation of iPSC-derived brain tumor models remains in its infancy, a majority of studies have concentrated their efforts to model the most aggressive forms of brain tumors, glioblastoma, and medulloblastoma.

### 3.1. Induced PSC-Derived Glioblastoma Models

The first human iPSC-derived gliomagenesis model was reported by Sancho-Martinez et al. in 2016 [29]. The model is generated by genetic manipulation of TP53 and dysregulating receptor tyrosine kinase (RTK) signaling in iPSC-derived NPCs, which is frequently seen in human gliomas. Upon orthotopic transplantation, the transformed NPCs resulted in aggressive human glioma-like tumors. Further screening for anticancer compounds identified three FDA-approved drugs specifically targeting the transformed cells in this model [29]. Earlier glioma models, including In vitro 2D or 3D culture systems and in vivo mouse models either genetically engineered (GEMMs) or patient-derived xenografts, have advanced our understanding of tumor initiation and progression. However, the inherent limitations associated with these models, ranging from their inability to replicate a normal human brain microenvironment due to their lack of intratumoral heterogeneity and cellular hierarchy, necessitate the development of other models that can be a better representative of in vivo tumor growth, progression, and response to treatment. Following the pioneering publication of Lancaster et al., several groups have used iPSC-derived brain organoids to phenocopy glioma features.

#### 3.1.1. Glioblastoma Organoids Using Genetically Engineered Brain Organoids

**Figure 3:**
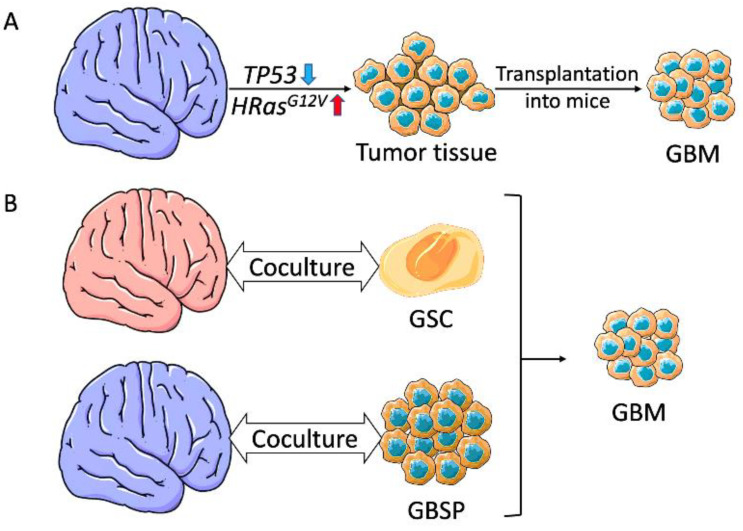
Modeling glioblastoma by genome editing and coculture. (**A**) Glioblastoma tumorigenesis by genome editing method. Cells from a brain organoid were edited by disruption of *TP53* and activating *HRas^G12V^*. These cells then fragmented the brain organoid into tumor tissue. Glioblastoma-like tumors were obtained after transplanting the tumor tissue into mice. (**B**) Glioblastoma tumorigenesis by coculture. Coculturing iPSC-derived brain organoids with patient-derived GSCs or mature brain organoids with glioblastoma spheroids induces glioblastoma generation. GBM, glioblastoma; GSC, glioma stem cell; GBSP, glioblastoma spheroid.

Using CRISPR/Cas9 technology of genome editing, Ogawa et al. mediated homologous recombination in a small number of cells in a 4-month-old mature brain organoid. This resulted in simultaneous disruption of the *TP53* gene and activation of the *HRas^G12V^* oncogene (Figure 3A). These mutations generated cells expressing glioblastoma (GBM) biomarkers with an invasive phenotype that destroyed the whole organoid structure and converted it into tumor tissue. Additionally, the transformed cells, when transplanted into mice, spawned tumors with glioblastoma-like features [112]. Bian et al. used a similar approach of genetic manipulation to generate a 3D glioblastoma model, which they called the neoplastic cerebral organoid (neoCOR). This tumor model was established by creating single or combined oncogenic mutations of clinically relevant genes via transposon- and CRISPR/Cas9-mediated mutagenesis. The resulting organoids displayed a glioblastoma signature capable of in vivo expansion and progression [113]. Although these methodologies can be deployed as a platform to monitor tumor initiation, they can hardly replicate tumor complexity, as they depend on genetic modification of certain genes that may not represent the actual glioma mutational profile.

#### 3.1.2. Glioblastoma Organoids Using Coculture Organoids

Coculture of iPSC-derived brain organoids with patient-derived glioma stem cells (GSCs) or neurospheres proved to be a better approach to model glioblastoma with complex genotypic and phenotypic heterogeneity (Figure 3B). The first to demonstrate this methodology was Ogawa et al. They cocultured mature brain organoids with spheroids of glioblastoma cells derived from the patient or created in the organoid by mutagenesis. While organoid-derived tumor cell spheres invaded 30% of the organoid after 24 days, the patient-derived GSC spheroids exhibited different invasion potential that was consistent with in vivo transplantation experiments [112]. This model provides a great framework to study glioblastoma progression, relapse after treatment, and novel therapeutics [114].

A glioma cerebral organoid model called GLICO (GLIoblastoma with Cerebral Organoids) was introduced by Linkous et al. This model was generated to replicate patient-specific glioblastomas in an attempt to customize treatment strategies to fit patients’ unique tumors. The authors cocultured different GFP-labeled GSCs derived from patients’ tumors with mature cerebral brain organoids. The resulting tumor cells proliferated, migrated, and integrated themselves within the brain organoid using different routes of microtubules based on the intrinsic properties of the invading GSC line. The GLICO model proved to be advantageous, as it recapitulated many features of in situ glioblastoma. It invaded and subsequently destroyed the organoid tissue, formed microtubules for communication and invasion as seen in in situ glioblastoma, and expressed key genetic features and signaling pathways [115]. This was further confirmed using single-cell RNA sequencing [27,116]. Comparing the transcriptomic profiles of tumor cells derived from patient-specific glioblastoma models (2D spheres, tumor organoids, GLICO, and patient-derived xenografts) showed that the GLICO model is the best representative of patients’ own primary tumors, highlighting the importance of the microenvironment in the maintenance of GSC cellular states and plasticity [116]. Strikingly similar to the primary tumors, the GLICO model was found to be enriched in GSCs with NPC-like and oligodendrocyte progenitor cell features and to differentially express markers related to stemness (SOX4), invasiveness (BCAN), and the NOTCH signaling pathway [116]. A recent study thoroughly analyzed the cell fate of GSCs as they invade the organoid in the GLICO model. Using fluorescent labeling of patient-derived GSCs, the authors monitored the process at early stages and realized the simultaneous existence of undifferentiated and differentiated GSCs, concomitant with Pine et al. [117]. Furthermore, GLICO has been the preferred glioblastoma platform for drug screening experiments due to its ease of production [11].

Lately, Goranci-Buzhala et al. compared the protocols described by Linkous and Ogawa with another method that generated a glioma model by simultaneous coculturing of GSCs and iPSCs, in an attempt to characterize GSC invasion patterns. They showed that GSCs tend to home to brain organoids of different maturity levels. This propensity is enhanced for mature brain organoids and varies between primary and recurrent GSCs. They also found that cocultures of GSCs and iPSCs do not reflect the in vivo process [118]. Although the coculture models of glioblastoma have surpassed other glioma organoid models in featuring tumor-normal brain microenvironment interaction, it still suffers from a lack of vascularization and immune cells.

#### 3.1.3. Other iPSC-Derived Models for Glioblastoma

Glioblastoma avatars derived from genome-edited human iPSCs have been shown to recapitulate the pathobiology of the disease. The study involved the introduction of GBM-associated genetic driver mutations into iPSCs, followed by their differentiation into NPCs. Finally, the edited NPCs were engrafted orthotopically in immunocompromised mice, forming GBM-like tumors. Moreover, cultures of dissociated tumor cells were found to possess cancerous phenotypes and to form, upon re-engraftment, secondary tumors with features similar to the patient samples [119].

Another glioblastoma modeling paradigm that used patient-derived iPSCs was recently reported. The authors deployed iPSCs from a patient with c-met mutation, a hallmark of glioblastoma, to generate a neuronal organoid simulating the disease. The c-met-mutated iPSCs developed neuron-like organoids that lasted up to one year with features characteristics of glioblastoma [120].

### 3.2. Induced PSC-Derived Medulloblastoma Models

Medulloblastoma (MB) is the most common malignant embryonal tumor accounting for 20% of all pediatric brain tumors [26]. According to an international consensus paper in 2012, medulloblastoma is divided into four molecular subgroups: WNT, SHH, Group 3, and Group 4 [121]. Subsequent studies reported additional intra-subgroup heterogeneity, especially within Group 3 and Group 4. A second-generation molecular subgrouping of Group 3/4 MB using DNA methylation and transcriptomic profiles has identified eight subtypes (I-VIII), each associated with distinct cytogenetic and clinicopathological features [122]. A multitude of genetic aberrations and heritable factors are known to drive medulloblastoma. Amplification of oncogenes, such as MYC and MYCN, is highly correlated with the disease and is recurrently altered in different subgroups [123,124]. Germline mutations in genes involved in developmental signaling pathways, such as APC (associated with familial adenomatous polyposis (FAP) syndrome), TP53 (linked to Li-Fraumeni syndrome), SUFU, and PTCH1 (manifestation of Gorlin syndrome) confer a significantly high risk of developing medulloblastoma [124]. With the limitations of available models and the unprecedented opportunities lent by iPSC-derived organoids in brain cancer, efforts are focused on exploiting iPSCs as a renewable cell source to study medulloblastoma initiation and progression.

#### 3.2.1. SHH-Medulloblastoma Models

The molecular classification of sonic hedgehog MB (SHH-MB) has identified constitutive activation of the SHH signaling pathway in patients. SHH-MB accounts for ~30% of all MB cases, with a majority being infants (<3 years of age) and adults (>16 years of age) [124,125]. Despite improved survival, current treatments have cognitive side effects that jeopardize the lives of affected children. Thus, it is an urgent need to seek other therapeutic strategies that can preserve a healthy brain. To accomplish this, reliable models are needed to generate faithful replicas of human MB that can be used to test effective treatments.

**Figure 4:**
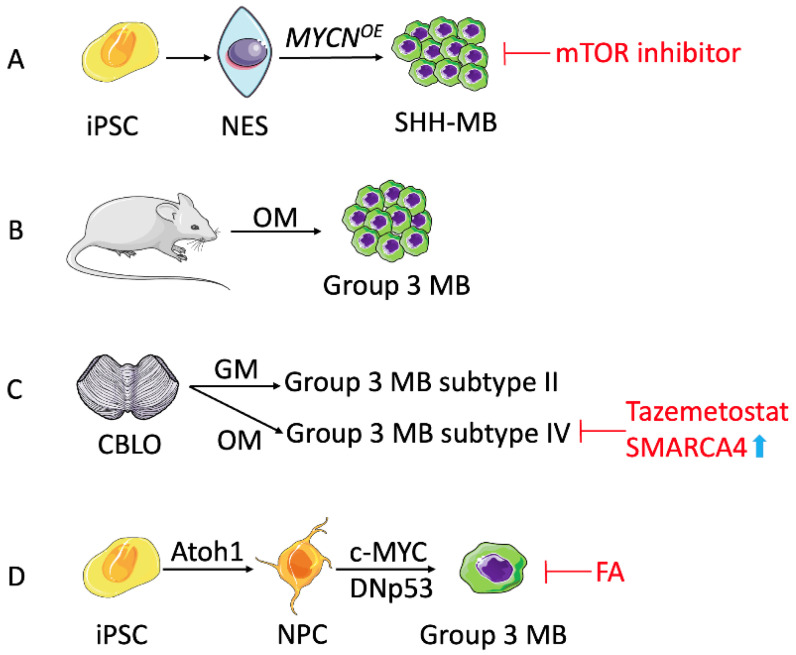
Generating SHH-medulloblastoma and Group 3 medulloblastoma tumor models. (**A**) SHH-MB induction from iPSCs. By transducing the MYCN gene into iPSC-derived NES (neuroepithelial stem) cells and then transplanting the NES cells into mice, SHH-MB tumors were produced. LGALS1 is highly expressed in both NES cells and SHH-MB tumor samples. mTOR inhibitor is a potential drug for SHH-MB. (**B**) Mice overexpressing OM developed Group 3 MB. (**C**) GM overexpression in iPSC-derived cerebellar organoids (CBLO) induced Group 3 MB subtype II, which had a higher risk than that of OM. Tazemetostat and SMARCA4 overexpression could inhibit OM-induced Group 3 MB. (**D**) Atoh1-coding mRNA induced rapid differentiation from iPSCs to NPCs. MYC overexpression and TP53 silencing made NPC differentiate into aggressive Group 3 MB, and frondoside A (FA) may rescue it. OM, OTX2+c-MYC overexpressing; GM, GFI1+c-MYC overexpressing.

Huang et al. used iPSCs to generate neuroepithelial stem (NES) cells with genetic tumor predisposition to study medulloblastoma [123]. Transduction of MYCN, a known driver of the disease, into iPSC-derived NES cells followed by orthotopic implantation in immunocompromised mice resulted in tumors clustered with SHH-MB based on DNA methylation and transcriptomic analyses (Figure 4A). Albeit MYCN-driven engineered mouse models were presented with medulloblastoma, stem-cell-derived tumors aligned most closely with the primary disease observed in patients. Another mutation, responsible for Gorlin syndrome (a tumor-prone condition) and predisposing patients to medulloblastoma, is in the PTCH1 gene, a member of the hedgehog signaling pathway. NESCs derived from iPSCs of patients with Gorlin syndrome bearing PTCH1 mutation displayed neural characteristics and the ability to recapitulate SHH medulloblastoma upon orthotopic engraftment in the murine model. Further genetic anomalies in Gorlin NES cells, including mutations in DDX3X and GSE1 genes, accelerated tumorigenesis and demonstrated cooperativity among these genes and PTCH1, further elaborating on the genetic causation of medulloblastoma [123].

In a similar approach, Čančer et al. developed humanized models of SHH-MB utilizing iPSC reprogramming and primary NES culturing. Ectopic overexpression of MYCN in NESCs derived from either iPSC or primary human hindbrain generated in vivo tumors with varying degrees of tumorigenicity. Although both models developed tumors with clinically relevant signatures of infant SHH-MB, the iPSC-derived tumors presented with a more aggressive phenotype. The authors attributed this increased malignancy to Oct4 upregulation and subsequent mTOR activation. This was validated by the fact that mTOR inhibitors effectively suppress viability, migration, and SHH-MB growth [126].

Another iPSC-derived medulloblastoma model was generated by Ikemoto et al. using fibroblasts from four patients with Gorlin syndrome to study the pathogenesis of the disease. One of the models exhibited somatic loss of heterozygosity of the PTCH1 wild-type allele, recapitulating the development of the SHH subgroup of medulloblastoma seen in patients with Gorlin syndrome carrying PTCH1 germline mutations [127].

Analogous to the previous studies, Susanto et al. described the formation of SHH-MB in mouse cerebellum following transplantation of NES cells reprogrammed from iPSCs of Gorlin syndrome patient skin cells with PTCH1 familial mutation. The model showed a progressive upregulation of inflammatory and epithelial-to-mesenchymal transition genes during primary and secondary tumor development, offering the possibility of a stepwise follow-up of tumor progression [125]. The authors also identified the LGALS1 gene, which codes for galectin-1, was highly expressed in primary and secondary NES cells as well as SHH tumor samples, suggesting an important role of this gene in medulloblastoma development [125].

#### 3.2.2. Group 3 Medulloblastoma Models

Group 3 medulloblastoma occurs in ~25% of all MB patients. It is characterized by poor clinical outcomes with a high incidence of metastasis at diagnosis and 5-year overall survival of <60% [124]. While MYC amplification (~20% of patients) is the hallmark feature of Group 3 MB, other driver gene alterations are reported in patients with this subtype, including overexpression of MYCN (5%) and OTX2 (3%), and somatic mutations in KBTBD4 (6%) SMARCA4 (9%), CTDNEP1 (5%), KMT2D (5%), and GFI1 (4%) [124,128]. Patients with Group 3 MB have the worst prognosis and suffer a harsh treatment regimen. Therefore, it is of paramount importance to develop a humanized model of this pediatric tumor to identify and screen for personalized drugs tailored to each patient’s genetic condition.

In a recent study, cerebellar organoids were exploited to create a medulloblastoma model simulating the Group 3 subtype. The authors reproduced in vivo the genetic aberrations identified in patients with Group 3 tumors. While mice overexpressing the gene combinations GFI1 + c-MYC (GM) and OTX2 + c-MYC (OM) induced brain tumors, only the latter combination developed medulloblastoma clustered with Group 3 subtype (Figure 4B), highlighting the role of OTX2 and c-MYC as the medulloblastoma driver genes. Using these genetic combinations, iPSC-derived cerebellar organoids with GM and OM gene amplification were developed to recapitulate Group 3 MB (Figure 4C). Interestingly, the GM tumors were categorized as subtype II, the high-risk Group 3 tumors, whereas OM tumors were categorized as subtype IV, the standard-risk Group 3 tumors, according to the DNA methylation profile and Group 3-specific markers. Furthermore, the study reported that SMARCA4 overexpression thwarted OM-induced Group 3 medulloblastoma in vivo and in cerebellar organoids. Its mutant, SMARCA4 T910M, counteracted the functions of the wild-type protein in medulloblastoma patients. Additionally, their medulloblastoma model showed sensitivity towards tazemetostat, an EZH2 inhibitor, suggesting it may be a potential drug for Group 3 medulloblastoma patients overexpressing OTX2 and c-MYC [129].

Lately, a synthetic mRNA-driven strategy was used to develop the MYC-driven medulloblastoma model mimicking the Group 3 subtype (Figure 4D). The synthetic mRNA coding for Atoh1, a proneural transcription factor, induced rapid differentiation of human iPSCs into neuronal precursors. Further transformation of these neuronal precursors to express the MYC oncogene and dominant negative TP53 resulted in aggressive tumors in the cerebella of immunocompromised mice recapitulating the histological and transcriptomic features of Group 3 MB [130]. This study also showed that frondoside A (FA), a naturally occurring marine compound, to be selectively cytotoxic against MYC-driven MB and to inhibit the expression of MYC and its downstream effector genes in MB cells. The FA antitumor potency positively correlated with MYC levels, presenting it as a potential drug for the treatment of cancers with MYC amplification [130].

### 3.3. Other Brain Tumor Models

Parisian et al. developed an inducible SMARCB1 loss-of-function system inside iPSC-derived cerebral organoids. SMARCB1 inactivation is strongly associated with aggressive atypical teratoid rhabdoid tumors (ATRT) in children. They showed the effect of SMARCB1 loss is dependent on neural differentiation stages, with a potential to drive cellular transformation during early stages of neural differentiation [131]. In terms of modeling benign neuroendocrine tumors, pituitary organoids could be generated from reprogrammed peripheral blood mononuclear cells of Cushing disease patients that harbored germline mutations. These organoids resembled similar adenoma characteristics of the tumor tissue [132].

Key studies modeling iPSC-derived malignant brain tumors are summarized in Table 1.

## 4. Conclusions and Future Perspectives

Induced PSCs have revolutionized biological and biomedical research for their remarkable renewal and differentiation potential. In neurodegenerative disease research, iPSCs are particularly important since they can be differentiated into brain-specific cell types and used in cell replacement therapy to restore brain function. Production of brain-specific cells is of great interest for generating isogenic brain organoids, BBB, and brain tumor models. At the same time, brain organoid and BBB models have widespread use in developmental studies, brain functional characterization, disease modeling, and drug screening. Concerning brain tumor modeling, iPSCs have attracted a great deal of research interest as they may be engineered to harbor specific mutations and be differentiated into different tumor types.

Brain tumor modeling from human iPSCs is superior for disease pathogenesis study because it possesses important species-specific attributes of human brain that are absent in animal models [133]. With the need for reliable human tumor models, renewable and scalable iPSC-based platforms are supplanting competing technologies such as genetically engineered mouse models (GEMMs) and patient-derived xenografts. While GEMMs are widely used because of their ability to accurately mimic tumor pathophysiology, morphological and physiological differences between human and rodent brains restrict their application [30]. Patient-derived xenografts have been the best platform to date to study brain tumors, but they failed to model tumor initiation and had poor scalability [113]. Induced PSC-derived platforms can compensate for these drawbacks. When combined with mouse models or patient-derived tumor tissue, iPSCs enhance the scalability of tumor models, the scope for tumor initiation and invasion studies, and the opportunity for drug testing [23].

Two major strategies are employed to obtain iPSC-derived brain tumor organoids: using iPSCs via genome editing technology to introduce driver mutations (e.g., neoCOR) or using patient-derived cells (e.g., GLICO). Fully iPSC-derived models such as neoCOR are excellent platforms to study neoplastic transformation mechanisms and tumor origin. They share similar transcriptomic profiles and invasive properties with the original tumor. However, their molecular features may be different from the natural tumor, and they may not retain intratumor heterogeneity [23]. Fully iPSC-derived models lack oxygen and nutrient supply owing to poor vascularization that affects cell survival and organoid cytoarchitecture [24]. These models also suffer from the absence of an immunological compartment and microenvironment of natural tumors [23]. Lack of vascularization might be confronted by coculturing organoids with endothelial cells, providing VEGF as a supplement, or inducing the expression of hETV2 [10]. Induced PSC-derived brain organoids might be complemented with patient- or iPSC-derived immune cells (e.g., TILs and microglia) to overcome the barriers concerning the immune landscape [23]. The genetic manipulation step poses a significant technical challenge in obtaining stably engineered cells for organoids [24]. As fully iPSC-derived brain tumor models currently offer limited scope for studying complex abnormalities, generating models incorporating more genetic alterations may be a future research direction worth exploring. Such models may be also utilized to evaluate the potential of genome editing technologies as mutation-correcting therapy.

Brain tumor organoids such as GLICO use patient-derived tumor cells to retain the heterogeneity, histopathology, and genetic makeup of tumor cells, but they still lack a native tumor microenvironment [134]. These models are especially useful to investigate invasion. When patient-derived tumor cells are combined with isogenic iPSC-derived brain cells, this forms the basis for establishing autologous invasion assays [24]. Though the concept faces challenges of robust reprogramming of patient-derived cells in a reasonable period, it can be an excellent system to characterize the invasiveness and drug resistance of individual tumors towards rational therapeutic planning and personalized medicine [24].

An important consideration on brain tumor organoid models is keeping them viable for a prolonged time, irrespective of their types [24]. Brain organoid models also lack reproducibility in the form of variation in defective gene expression, input–output system, and desired spatial orientation. These may be addressed by the use of microfluidic technology to control local stimuli, in vivo transplantation, and bioengineered scaffolds, respectively [135]. To support months-long experiments with cultured organoids, improved bioreactor designs are needed to reduce the possibility of contamination or malfunction [136]. An appealing alternative to culture organoids might be 3D bioprinted organoids containing all the components of a brain tumor and its microenvironment and maintaining proper cell–cell and cell–matrix interaction. However, the overwhelming complexity of brain tumors and high cost has restricted their use [23,134].

Despite considerable success in glioma and medulloblastoma modeling, meningioma and ependymoma, two main types of brain tumors, have not been modeled using the pluripotent stem cell approach. Meningioma originates from the meninges, while ependymoma involves ventricle-lining ependymal cells. A potential use of iPSCs involves organoid formation for these tumors. Apart from disease modeling, iPSC-derived brain organoid models can be exploited for neurotoxicological studies, such as understanding the neurotoxic mechanism of antineoplastic drugs or carcinogenicity potency of environmental agents (heavy metals, background radiation, etc.).

Brain organoid models could also be useful for faster clinical decision-making at a single-patient level via biobanking, where the genetic features of a patient’s tumor could be matched against a collection of drug-sensitive organoids. Primary tumor-derived organoids are the most relevant mimetics in this direction, as they retain heterogeneous cancer stem cell composition to ensure their simultaneous growth and desired cellular hierarchy [137,138]. Co-clinical trials are increasingly supporting the similarity of cancer drug responses in biobanked organoids and patients [134]. These models hold promise to become excellent systems for semi-high-throughput drug screening in the foreseeable future [23,24]. With the limitations of brain tumor models resolved in the coming years through science and technology advancement, their full potential in disease modeling and drug discovery can be realized.

## Figures and Tables

**Table 1:** Induced PSC-derived malignant brain tumor models and their features.

Brain Tumor	Author	Model	Features	Key Findings
Glioblastoma	Sancho-Martinez et al. [29]	Orthotopic engraftment of engineered NPCs in mice	*TP53^-/-^, RAS^OE^/EGFR^OE^/SRC^OE^*	Engineered NPCs acquired glioma features. Transplantation yielded aggressive tumors.
	Ogawa et al. [112]	Engineered tumor organoid orthotopically engrafted in mice	*HRas^G12V^/TP53^-/-^*	Engineered cells expressed GBM markers and invasive phenotype.
	Bian et al. [113]	Engineered tumor organoid	*MYC^OE^, CDKN2A^–/–^/CDKN2B^–/–^/EGFR^OE^/EGFRvIII^OE^, NF1^–/–^/PTEN^–/–^/TP53^–/–^, EGFRvIII^OE^/PTEN^–/–^/CDKN2A^–/–^*	GBM organoids were capable of in vivo expansion and progression.
	Ogawa et al. [112]	Engineered tumor cell line/patient-derived glioblastoma cell line transplanted into cerebral organoid	*HRas^G12V^/TP53^-/-^* (Engineered tumor cell line)	Patient-derived cells showed different invasion potential than that of organoid-derived tumor cell spheres.
	Linkous et al. [115]	Organoid cocultured with GSCs	-	Patient-specific GBM could be studied ex-vivo. Scalable GLICO provided a reliable phenocopy of patient GBM.
	Goranci-Buzhala et al. [118]	Hybrid organoids by coculturing iPSCs with GSCs	-	3D GSC invasion assays were developed.
	Hwang et al. [120]	Engineered tumor organoid	*c-met^OE^*	iPSC aggregates displayed genomic network and phenotype of primary human GBM. Organoids were sensitive to temozolomide.
	Koga et al. [119]	Orthotopic engraftment of engineered NPCs in mice	*NF1^–/–^/PTEN^–/–^,* *TP53^-/-^/PDGFRA^Δ8−9^*	GBM model contained intra- and inter-tumor heterogeneity and provided platform for assessment of tumor development.
Medulloblastoma	Ballabio et al. [129]	Engineered tumor organoid	*GFI1^OE^ /c-MYC^OE^, OTX2^OE^ /c-MYC^OE^*	*OTX2* and *c-MYC* were identified as strong Group 3 MB drivers. SMARCA4 expression and tazemetostat negate *OTX2/c-MYC* tumorigenesis.
	Huang et al. [123]	Orthotopic engraftment of engineered/Gorlin NESCs in mice	*MYCN^OE^, PTCH1^+/−^*	Engineered NESCs mimicked tumor subtype and epigenetic profile. Gorlin NESCs retained MB predisposition.
	Čančer et al. [126]	Orthotopic engraftment of engineered NESCs in mice	*MYCN^OE^*	Aggressive SHH-MB with mTOR activation and increased Oct4
	Ikemoto et al. [127]	Gorlin iPSCs subcutaneously implanted in mice	Heterozygous *PTCH1* mutations	Gorlin iPSCs developed MB with secondary somatic *PTCH1* mutations.
	Susanto et al. [125]	Orthotopic engraftment of Gorlin NESCs in mice	*PTCH1* 1762insG	Gorlin NESCs formed tumors mimicking human SHH-MB.
	Xue et al. [130]	Orthotopic engraftment of engineered NPCs in mice	c-MYC/DNp53 coexpression	MYC-driven tumors recapitulated group 3 MB.

OE, overexpression; GSC, glioma stem cell; GBM, glioblastoma; GLICO, glioma cerebral organoid; NPC, neural progenitor cell; MB, medulloblastoma; NESC, neuroepithelial stem cell.

## Data Availability

The datasets generated and used/or analyzed are published in this paper, and available from the corresponding author upon request.
